# Propensity Scores for Prediction and Characterization of Bioluminescent Proteins from Sequences

**DOI:** 10.1371/journal.pone.0097158

**Published:** 2014-05-14

**Authors:** Hui-Ling Huang

**Affiliations:** Institute of Bioinformatics and Systems Biology, National Chiao Tung University, Hsinchu, Taiwan; Department of Biological Science and Technology, National Chiao Tung University, Hsinchu, Taiwan; CSIR-Institute of Microbial Technology, India

## Abstract

Bioluminescent proteins (BLPs) are a class of proteins with various mechanisms of light emission such as bioluminescence and fluorescence from luminous organisms. While valuable for commercial and medical applications, identification of BLPs, including luciferases and fluorescent proteins (FPs), is rather challenging, owing to their high variety of protein sequences. Moreover, characterization of BLPs facilitates mutagenesis analysis to enhance bioluminescence and fluorescence. Therefore, this study proposes a novel methodological approach to estimating the propensity scores of 400 dipeptides and 20 amino acids in order to design two prediction methods and characterize BLPs based on a scoring card method (SCM). The SCMBLP method for predicting BLPs achieves an accuracy of 90.83% for 10-fold cross-validation higher than existing support vector machine based methods and a test accuracy of 82.85%. A dataset consisting of 269 luciferases and 216 FPs is also established to design the SCMLFP prediction method, which achieves training and test accuracies of 97.10% and 96.28%, respectively. Additionally, four informative physicochemical properties of 20 amino acids are identified using the estimated propensity scores to characterize BLPs as follows: 1) high transfer free energy from inside to the protein surface, 2) high occurrence frequency of residues in the transmembrane regions of the protein, 3) large hydrophobicity scale from the native protein structure, and 4) high correlation coefficient (*R* = 0.921) between the amino acid compositions of BLPs and integral membrane proteins. Further analyzing BLPs reveals that luciferases have a larger value of *R* (0.937) than FPs (0.635), suggesting that luciferases tend to locate near the cell membrane location rather than FPs for convenient receipt of extracellular ions. Importantly, the propensity scores of dipeptides and amino acids and the identified properties facilitate efforts to predict, characterize, and apply BLPs, including luciferases, photoproteins, and FPs. The web server is available at http://iclab.life.nctu.edu.tw/SCMBLP/index.html.

## Introduction

Bioluminescent proteins (BLPs) are a class of proteins with various mechanisms of light emission such as bioluminescence and fluorescence from luminous organisms. Bioluminescence of organisms is the emission of visible light by a natural chemical reaction in diverse forms of morphology within a living organism [1]. In the bioluminescence process, the luciferin-luciferase reaction involves two chemicals, luciferin and luciferase. Luciferins are a class of small light-emitting heterocyclic compounds that are oxidized in the presence of the enzyme luciferase. Luciferase generally refers to the class of oxidative enzymes that catalyzes the oxidation of luciferin to emit light with an intermediate called oxyluciferin. Luciferins typically undergo enzyme-catalyzed oxidation, and the unstable oxyluciferin emits light upon decaying to its ground state. The amount of light emitted is proportional to the concentration of the substrate luciferins. Firefly luciferin is a well-studied luciferin found in many firefly species, in which oxygen, ATP, and magnesium ions are required for light emission [2].

As a stable protein complex, photoprotein consists of a catalyzing protein (i.e. a luciferase variant) and factors required for light emission including the luciferin and oxygen. Photoproteins do not exhibit a luciferin-luciferase reaction and emits light without the intervention of an enzyme. Photoproteins are triggered to produce light upon binding with another ion or co-factor such as Ca^2+^, causing a conformational change in the protein. Photoproteins referring to luciferase-like proteins display luminescence proportional to the amount of the catalyzing protein. The number of photoproteins with known structures is relatively small, compared with that of BLPs. As a photoprotein isolated from luminescent jellyfish *Aequorea victoria*, aequorin has been used to measure calcium ion concentration. Aequorin consists of two distinct units, the apoprotein apoaequorin and a latently luminescent molecule coelenterazine, i.e. a luciferin. When a calcium ion Ca^2+^ binds to aequorin, the protein complex decomposes into apoaequorin, coelenteramide and CO_2_, accompanied by the emission of light [3].

Rather than producing their own light, fluorescence molecules absorb photons, which temporarily excite electrons to a higher energy state. When relaxing rapidly to their ground state, the electrons rerelease their energy, usually at a longer wavelength. Fluorescent proteins (FPs) belong to a structurally homologous class of proteins that share the unique property of being-sufficient to form a visible chromophore from a sequence of three amino acids within their own polypeptide sequence [4]. The tightly packed nature of a barrel excludes solvent molecules, thereby protecting the chromophore fluorescence from quenching by water. Fluorescent proteins are well established markers for gene expression and protein targeting in intact cells and organisms. The mechanism of light production through a chemical reaction distinguishes bioluminescence from fluorescence [4]. The green fluorescent protein (GFP) from jellyfish *Aequorea victoria* can be colocalized with its bioluminescence counterpart (i.e. aequorin).

BLPs serve in a variety of functions of cellular processes. With the increasing number of innovative commercial and medical applications using BLPs, understanding BLPs provides fascinating challenges for fundamental sciences and numerous opportunities for practical applications. Prediction and characterization of BLPs are of priority concern in a variety of research fields. Despite the availability of experimental methods to investigate BLPs [5], such methods are often time-consuming and severely limited in scope, leading to a lack of large datasets of reviewed BLPs. Therefore, developing computational methods to predict and characterize BLPs, including luciferases and FPs, from a sequence is more challenging, owing to their high variety in the protein sequence and lack of a large BLP dataset. The sequence-based prediction method can fast identify putative BLPs from sequences with/without known structure information for further validation.

By establishing a dataset consisting of 441 BLPs and 18,502 non-BLPs, Kandaswamy *et al*. [6] proposed a computational method (known as BLProt) to predict BLPs. BLProt is trained using a dataset consisting of 300 BLPs and 300 non-BLPs and, then, evaluated by an extremely imbalanced test dataset (141 BLPs and 18,202 non-BLPs). BLProt uses a support vector machine (SVM) and 100 physicochemical properties selected by adopting three feature selection methods. BLProt achieves an accuracy of 80.0% for 5-fold cross-validation (5-CV) from training and an accuracy of 80.06% from testing. Huang *et al*. [7] subsequently proposed an improved SVM-based method (known as PBLP), in which 15 physicochemical properties are used to predict BLPs; in addition, the training (5-CV) and test accuracies are 84.50% and 81.79%, respectively. Zhao *et al*. [8] developed a BLPre method with a training accuracy of 85.17% for 10-CV to predict BLPs by using a model based on position specific scoring matrix (PSSM) and auto covariance. Fan and Li [9] recently proposed an accurate SVM-based method (known as SVM-Hybrid) by integrating multiple features, including dipeptide composition, reduced amino acid composition, PSSM, and auto covariance of averaged chemical shift. SVM-Hybrid achieves a training accuracy of 90.50% for 10-CV. Although yielding an acceptable accuracy, existing SVM-based prediction methods suffered from obtaining human-interpretable knowledge for further understanding BLPs.

Predicting and characterizing BLPs as well as luciferases and FPs in general conditions from a sequence are worthwhile yet challenging tasks. This study proposes a novel methodological approach to estimating the propensity scores of 400 dipeptides and 20 amino acids in order to design two prediction methods, i.e. SCMBLP and SCMLFP, based on a scoring card method (SCM) [10], [11]. In addition to using the existing datasets of BLPs for comparison, this study also establishes a balanced dataset consisting of 274 non-BLPs and 274 BLPs, including 141 BLPs from seed proteins of the Pfam database, 94 BLPs using the GO term GO: 0008218 “bioluminescence”, and 39 BLPs using the keyword “photoprotein” from the Protein Data Bank (PDB database) to evaluate SCMBLP. According to those results, SCMBLP achieves an accuracy of 90.83% for 10-CV, better than the existing SVM-based methods, and the test accuracy of 82.85%. Additionally, a dataset of 269 luciferases and 216 FPs is established to design a novel SCMLFP method for distinguishing luciferases from FPs. Evaluation results indicate that the SCMLFP method yields training and test accuracies of 97.10% and 96.28%, respectively. This study also identifies informative physicochemical properties from the AAindex database [12], [13] by using propensity scores of 20 amino acids to gain further insight into BLPs, luciferase, photoproteins, and FPs. Results of this study demonstrate that the propensity scores of dipeptides and amino acids and the identified properties greatly facilitate mutagenesis analysis to enhance the bioluminescence and fluorescence of BLPs.

## Materials and Methods

**Figure 1:**
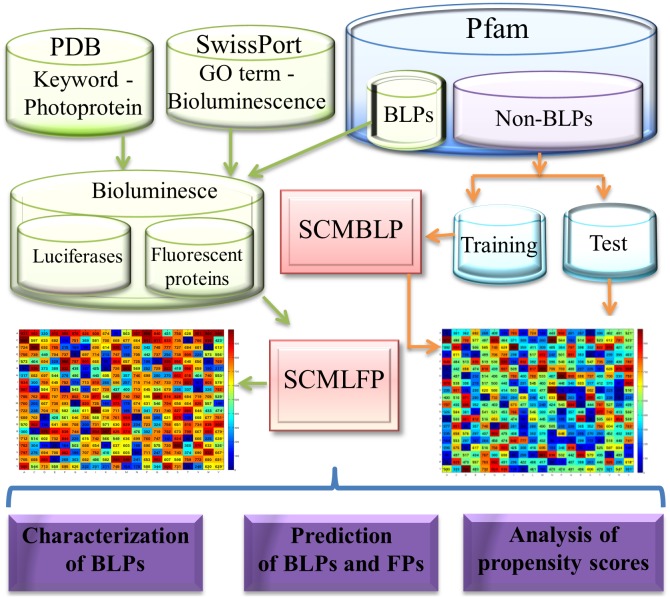
The flowchart of system designs for predicting and characterizing bioluminescent proteins (BLPs). The SCMBLP method predicts BLPs and the SCMLFP method distinguishes luciferases from fluorescent proteins. The obtained scoring cards are used to further analyze BLPs.

This study proposes a novel methodological approach to estimating the propensity scores of 400 dipeptides and 20 amino acids to design two prediction methods, i.e. SCMBLP and SCMLFP, based on a scoring card method (SCM). Figure 1 shows the flowchart of system designs involving datasets, methods, and analysis of propensity scores to predict and characterize BLPs.

### Datasets

While obtaining 300 BLPs from seed proteins of the Pfam database [14], Kandaswamy *et al*. [6] enriched this dataset consisting of 441 BLPs with sequence identity < = 40%. The used training set, known as BLP-TRN, consists of 300 BLPs selected from the 441 BLPs and 300 non-BLPs from seed proteins of Pfam protein families that can be obtained from this work [6]. The test dataset used in the studies [6]–[9] consists of 141 BLPs and 18202 non-BLPs, which is an extremely imbalanced dataset. After manual examination by inquiring the domain classification in the Pfam database for each BLP, the dataset of 441 BLPs consist of three groups: 269 luciferases, 84 FPs, and 88 others (without obviously unique categorization into luciferase or FP). Owing to the extreme imbalance of the test dataset, the overall accuracy only is an unsatisfactory performance index used in related studies [6]–[9]. This study also established a balanced dataset, known as BLP-TEST, consisting of 274 non-BLPs (randomly selected from the 18,202 non-BLPs) and 274 BLPs including the 141 BLPs from seed proteins of the Pfam database, 94 BLPs from 44 species using the GO term GO:0008218 “bioluminescence” annotated on SwissProt, and 39 BLPs (without duplicated sequences) using the keyword “photoprotein” from the PDB database.

To investigate the properties of discriminating between luciferases and FPs from a sequence, a dataset consisting of 269 luciferases and 216 FPs was created (given in Files S1 and S2, respectively). The 269 luciferases were obtained from the 441 BLPs in the Pfam database. There are 512 sequences obtained by using the keyword “fluorescent protein” from the PDB database. Only the 189 sequences with the classification category “fluorescent protein” were adopted. Deleting the duplicated sequences among the 189 and 84 FPs (from the Pfam database) led to a final dataset of 216 FPs. The luciferases and FPs in the dataset were randomly divided into ten groups. Among which, nine groups were used for training (i.e. LFP-TRN) and the other one for testing (i.e. LFP-TEST) in turn to design the SCMLFP method. Therefore, 10 independent training and test datasets are available to evaluate SCMLFP.

This study also calculates the Pearson’s correlation coefficients (the *R* values) between the propensity scores of amino acids to be a BLP and the physicochemical properties of amino acids in the AAindex database. The range of *R* is [–0.733, 0.592]. Table 4 lists four physicochemical properties of amino acids with a large value of *R* estimated in a general condition. Figure 5 shows the correlations of propensity scores and the four identified properties of 20 amino acids. The four properties of interest with high absolute values of *R* are discussed below.

### Scoring Card Method

The scoring card method (SCM) [10], [11] is a general-purpose method for predicting and analyzing protein functions from primary sequences by estimating propensity scores of 400 dipeptides and 20 amino acids to be the protein with the investigated function. To apply the SCM method for designing a SCM-based predictor, the procedure mainly comprises the following steps: 1) both positive and negative datasets are prepared as input, 2) an initial scoring card with 400 propensity scores of dipeptides is generated using a statistical method, 3) propensity scores of 20 amino acids are derived from those of 400 dipeptides, 4) the scoring card is refined using a global optimization method, and 5) a binary SCM classifier with a threshold value is established. The algorithm of the SCMBLP method, based on SCM for predicting BLPs, is described below. Design of the SCMLFP method resembles that of SCMBLP by simply replacing the training dataset BLP-TRN with LFP-TRN. Further details of the SCM method and its applications can be found in these studies [10], [11].

Step 1: Adopt a training dataset BLP-TRN, which consists of two subsets for positive (BLP) and negative (non-BLP) datasets.

Step 2: Generate an initial scoring card, which consists of propensity scores of 400 dipeptides by using a statistical method as follows:

- Calculate the dipeptide composition of BLPs and non-BLPs from the positive and negative datasets;
- Obtain the propensity score of each individual dipeptide by subtracting the composition value of the dipeptide in non-BLPs from that of the dipeptide in BLPs; and.
- Normalize the scores of all dipeptides into the range [0, 1000].

Step 3: Calculate the propensity score of each amino acid O by averaging the 40 scores of dipeptides OX and XO where X can be any amino acid.

Step 4: Optimize the scoring card (referred to herein as Scard) of dipeptides, which consists of 400 scores by using an intelligent genetic algorithm (IGA) [15]. In the chromosome representation, the 400 real-valued variables within the range [0, 1000] are encoded into a chromosome of IGA. The fitness function of IGA is to maximize the prediction accuracy in terms of the area under the ROC curve (AUC) [16] and also the Pearson’s correlation coefficient (the *R* value) between the initial and optimized propensity scores of 20 amino acids. To avoid overfitting, the fitness function is calculated using a 10-CV assessment [10]. The fitness function is as follows (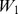 = 0.9 and 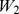 = 0.1 in this study).
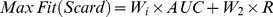
(1)


Step 5: Predict a sequence *P* bases on the scoring function *S(P)*, i.e. a weighted-sum score, and a threshold value determined by maximizing the prediction accuracy of using the training dataset.
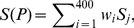
(2)where 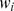 and 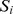 denotes the composition and propensity score of the *i*-th dipeptide, respectively. Additionally, *P* is classified as the positive class (i.e. BLP) when *S(P)* is greater than the threshold value; otherwise, *P* is the negative class (i.e. non-BLP).

### Identifying Informative Physicochemical Properties

Physicochemical properties of amino acids are well recognized to be effective features for predicting and analyzing protein functions from primary sequences [7], [12], [17]. Kandaswamy *et al*. [6] and Huang *et al*. [7] developed prediction methods for BLPs using SVM and a set of physicochemical properties selected from the AAindex database [13]. Since the mathematical vectors representing the 544 physicochemical properties in AAindex are distributed diversely, a set of properties can achieve a high prediction accuracy for binary classifier problems if the feature selection method is satisfactory, especially when using optimal feature selection approaches [7], [12], [17].

To characterize BLPs from the aspect of physicochemical properties of amino acids, this study proposes a novel method in which the properties of particular interest are identified, based on the scoring card of SCM while considering two factors. First, many physicochemical properties of amino acids in AAindex are investigated in a specific condition. Second, bioluminescence of organisms occurs in diverse forms of morphology with various mechanisms of light emission, which also heavily depends on environmental conditions. Therefore, this study elucidates informative physicochemical properties in a generalized condition to discriminate between BLPs and non-BLPs, as well as luciferases and FPs.

The propensity scores of 400 dipeptides and 20 amino acids to be a BLP obtained from the above-mentioned SCMBLP classifier greatly facilitate efforts to understand BLPs. The procedure to identify informative physicochemical properties is described below. First, whether or not the bioluminescence is a global property of sequence for general BLPs is examined. The examination method analyzes the distribution of locations of high-score dipeptides on the BLP and non-BLP sequences [8], [9]. A situation in which the high-score dipeptides are evenly distributed on the sequences implies that the bioluminescence is a global property of a sequence; otherwise, bioluminescence may occur in specific segments. Second, the procedure identifies the candidate physicochemical properties in AAindex with a large Pearson’s correlation coefficient between the properties and the propensity scores or composition of 20 amino acids. The candidate physicochemical properties investigated in a generalized condition are preferred for further analysis.

## Results and Discussion

### Propensity Scores of BLPs

**Figure 2:**
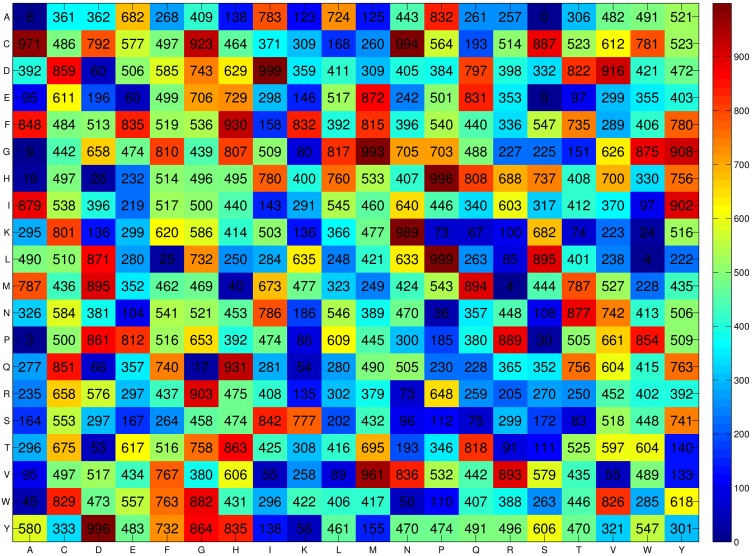
Heat map of the propensity scores of 400 dipeptides obtained from the SCMBLP method. Color bar is obtained using Jet of Matlab.

Figure 2 shows the propensity scores of 400 dipeptides to be a BLP obtained by using the SCMBLP method with the BLP-TRN dataset consisting of 300 BLPs and 300 non-BLPs. The propensity scores of 20 amino acids can be derived from the propensity scores of 400 dipeptides. Table 1 lists the propensity scores of amino acids to be a BLP and the amino acid compositions of BLPs and non-BLPs. The total number of amino acids in the used datasets of BLPs and non-BLPs is 130,904 and 120,607, respectively. The propensity scores and amino acid composition can be analyzed to further gain insight into how to characterize BLPs.

**Table 1:** The propensity scores of amino acids to be a bioluminescent protein (BLP) and amino acid composition (%) using BLP-TRN.

Amino acid	BLP Score (rank)	Composition of BLP: A (%)	Composition of Non-BLP: B (%)	Composition difference: A–B (%)
G-Gly	573.025 (1)	7.53	6.49	1.04
C-Cys	572.850 (2)	2.09	1.28	0.81
F-Phe	548.075 (3)	4.58	3.90	0.68
H-His	534.450 (4)	2.75	2.33	0.42
Y-Tyr	508.750 (5)	3.56	3.05	0.51
D-Asp	498.100 (6)	5.82	5.55	0.27
M-Met	483.150 (7)	2.42	2.27	0.15
V-Val	477.775 (8)	6.60	6.45	0.15
P-Pro	472.950 (9)	4.99	4.91	0.08
I-Ile	456.525 (10)	5.60	5.66	–0.05
T-Thr	452.750 (11)	5.51	5.40	0.11
N-Asn	451.125 (12)	4.23	4.21	0.02
Q-Gln	435.025 (13)	3.70	4.03	–0.33
W-Trp	434.575 (14)	1.22	1.27	–0.05
L-Leu	426.700 (15)	9.31	9.70	–0.39
E-Glu	404.075 (16)	6.25	6.79	–0.54
R-Arg	384.875 (17)	4.96	5.51	–0.55
S-Ser	368.500 (18)	6.61	7.27	–0.66
A-Ala	359.750 (19)	7.12	8.15	–1.03
K-Lys	336.275 (20)	5.14	5.80	–0.66
R	1.00	–0.30	–0.50	0.97

The total number of amino acids in the used datasets of BLPs and non-BLPs is 130,904 and 120,607, respectively.

Closely examining the dipeptide composition (not shown) reveals that the 20 top-ranked dipeptides according to propensity score (DI, LP, HP, YD, CN, GM, KN, CA, VM, QH, FH, CG, DV, GY, RG, IY, LS, MD, MQ, VR in order) make up BLPs (5.23%) and non-BLPs (4.37%). There are 19 individual dipeptides (except for IY) in which their compositions in BLPs are larger than those in non-BLPs. Compositions of the dipeptide IY for BLPs (0.1738%) and non-BLPs (0.1779%) are very close. The results indicate that BLPs have a larger number of dipeptides with high propensity scores than non-BLPs. Similarly, BLPs have a small number of dipeptides with low propensity scores. This result reveals that the propensity scores of dipeptides can discriminate between BLPs and non-BLPs.

The set of hydrophobic residues is {Ala, Ile, Leu, Met, Phe, Val, Cys, Gly}, and the other residues are hydrophilic residues according to the work [18]. The three top-ranked amino acids according to propensity scores are Gly = 573.025, Cys = 572.850, and Phe = 548.075, which belong to the class of hydrophobicity. The three amino acids with the smallest scores are Lys = 336.275, Ala = 359.750, and Ser = 368.500. The three hydrophobic residues Gly, Cys, and Phe make up BLPs (14.20%) and non-BLPs (11.67%). Based on the composition differences between BLPs and non-BLPs, the three individual amino acids Gly, Cys, and Phe are ranked at 1, 2, and 3, respectively. The top-10 high-score residues of BLPs have nine residues (except for Ile) with positive composition differences. Table 1 lists the compositions of BLPs and non-BLPs for residue Ile, which are 5.60% and 5.66%, respectively. The correlation coefficient *R* equals to 0.97 between the propensity scores of amino acids and composition difference of amino acids between BLPs and non-BLPs. Analysis results of amino acid composition indicate that BLPs have more hydrophobic residues and less hydrophilic residues than non-BLPs. Moreover, the high correlation coefficient suggests that the propensity scores of amino acids can also discriminate between BLPs and non-BLPs.

From the propensity scores of dipeptides and amino acids, we can infer that two individual amino acids with high propensity scores do not necessarily form a dipeptide with a high propensity score. For example, although the two top-ranked amino acids are Gly and Cys, the dipeptides Gly-Cys and Cys-Gly have propensity scores of 442 and 923, respectively. The propensity scores of two dipeptides with the same two residues are not the same, owing to the different dipeptide compositions of BLPs. It is hypothesized that the charged polar residues play an important role in placing the luciferin and chromophore in the right orientation facing the hydrophobic surface of substrate-binding cavity [19], resulting in the asymmetry scores of dipeptides. Notably, the dipeptide with the smallest score is Ala-Ser with a score 0. The score of Ser-Ala is also small, which equals to 164. The two residues Ala and Ser have small propensity scores, ranking 19 and 18, respectively.

### Prediction Accuracy of SCMBLP

**Figure 3:**
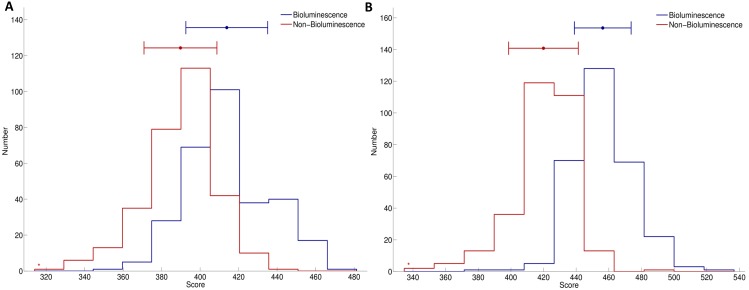
The histogram of propensity scores for BLPs and non-BLPs in the training dataset BLP-TRN. (A) Sequence scores before optimization (B) Optimized sequence scores.

The propensity score of a sequence to be a BLP is useful for discrimination between PLPs and non-BLPs. The histogram of sequence scores for BLPs and non-BLPs in the training dataset is given in Fig. 3. The distribution range of sequence scores for BLPs is reduced and shifted to the end of high score after the optimization on propensity scores. Furthermore, the distributions for BLPs and non-BLPs are more separable after optimization. A higher score of a sequence implies a larger probability that the sequence is a BLP.

This study also compares SCMBLP with the existing SVM-based methods [6]–[9] using the promising features: dipeptide composition (DPC), physicochemical properties (PCPs), PSSM and hybrid feature set. The SVM-DPC method using SVM with the DPC features is implemented for comparison. Owing to the extreme imbalance of the test dataset (i.e. 141 BLPs and 18,202 non-BLPs; 99.23% for non-BLP), the test accuracy heavily depends on the specificity performance of using the training dataset. If the design of the prediction methods tends to have a higher specificity performance than sensitivity performance (i.e. preference towards non-BLP), the test accuracy is easily higher than 90%. Therefore, it is not fair to evaluate the predictors using the test accuracy only. The training accuracy of 5-CV or 10-CV was commonly used for performance comparisons in these studies [6]–[9]. The comparisons of SCMBLP with existing methods using the training dataset BLP-TRN are given in Table 2.

**Table 2:** The comparisons of SCMBLP with some comparable methods using BLP-TRN in terms of 5- or 10-fold cross-validation accuracy (%).

Method	Classifier	Features	Optimization	Sensitivity	Specificity	Accuracy
BLProt [6]	SVM	100 PCPs	No	74.47^a^	84.21^a^	80.00
PBLP [7]	SVM	15 PCPs	Yes	84.11	79.25	84.50
BLPre [8]	SVM	PSSM-AC	No	79.33	91.00	85.17
SVM-Hybrid [9]	SVM	DPC+PSSM+RAAC+ACS	No	88.33	92.67	90.50
SVM-DPC	SVM	DPC	No	82.00	85.00	83.50
SCMBLP	SCM	DPC	Yes	89.67	92.00	90.83

RAAC: Reduced amino acid composition;

ACS: Average chemical shift;

DPC: Dipeptide composition.

a The results are test accuracies that the cross-validation accuracies were not reported.

Above comparisons reveal that the best SVM-based method is SVM-Hybrid with an accuracy of 90.50% for 10-CV using a hybrid feature set by integrating DPC, RAAC, PSSM, and auto covariance of average chemical shift (ACS) [9]. That SVM-DPC has an accuracy of 83.50% for 10-CV further reveals that DPC is a more effective feature type than PSSM and PCPs in terms of predicting BLPs. The proposed SCMBLP method of using SCM and DPC (90.83%) is superior to all the SVM-based methods. The high performance of SCMBLP arises mainly from the used optimization approach to adjusting 400 propensity scores of dipeptides, i.e. 400 features for classification. Owing to the difference in the feature number and optimization usage used, evaluating the effectiveness of individual types of features is relatively difficult. Based on the above results, we can conclude that the feature of dipeptides is effective in predicting BLPs, and the SCM classifier is comparable to the high-performance SVM classifier. Moreover, SVM is satisfactory in designing an accurate predictor and SCM is appropriate for analyzing the investigated functions with an acceptable accuracy from a sequence. However, the dipeptide features with propensity scores are more easily interpreted and helpful for subsequent analysis [10], [11].

To evaluate SCMBLP using test performance for observing the generalization ability, a balanced dataset consisting of different resources for comparison and analysis is created. The test dataset BLP-TEST consists of 274 non-BLPs and 274 BLPs. Table 3 summarizes the performance of SCMBLP on BLP-TEST. From Table 3, the following observations are made. First, the overall test accuracy is 82.85% with a sensitivity of 85.40% and a specificity of 80.29%. The threshold value of sequence scores used is 439.63, and can be adjusted according to the preference of a decision marker on the sensitivity and specificity. Second, while considering the training accuracy of 90.83% and the test accuracy of 82.85%, not-severe overtraining seems to occur. This overtraining can be alleviated by way of increasing the size of the training dataset and including all domains of luciferases and FPs with a sufficient number of samples. Third, the accuracies of BLPs from PDB, SwissProt, and the Pfam database are 94.87%, 86.17%, and 82.27%, respectively. The prediction accuracies on the datasets consisting of reviewed BLPs from PDB and SwissProt are larger than that of using seed proteins from the Pfam database. The 39 BLPs from the PDB database and 94 BLPs from SwissProt with their sequence scores are given in Tables S1 and S2, respectively.

**Table 3:** The test performance (%) of SCMBLP on various data sets.

Dataset (Source)	BLPs (PDB)	BLPs (SwissProt)	BLPs (Pfam)	Non-BLP (Pfam)	Total
Total no	39	94	141	274	548
True positive	37	81	116	220	454
Accuracy (%)	94.87	86.17	82.27	80.29	82.85

The overall accuracy is 82.85% with a sensitivity of 85.40% and a specificity of 80.29%.

**Table 4:** The propensity scores of amino acids to be a bioluminescent protein (BLP) and four physicochemical properties of amino acids with Pearson’s correlation coefficient (the *R* value).

Amino acid	BLP Score (rank)	Transfer free energy	Membrane preference	Hydrophobicity scale	Composition in nuclear proteins
G-Gly	573.025 (1)	0.3	1.08	–0.1	6.3
C-Cys	572.850 (2)	0.9 (1)	1.60 (1)	1.9 (1)	1.6 (2)
F-Phe	548.075 (3)	0.5	1.46	1.0	2.7
H-His	534.450 (4)	–0.1	1.00	0.4	2.1
Y-Tyr	508.750 (5)	–0.4	0.89	0.5	2.4
D-Asp	498.100 (6)	–0.6	0.27	–1.4	4.7
M-Met	483.150 (7)	0.4	1.52	0.5	2.3
V-Val	477.775 (8)	0.6	1.33	0.7	5.3
P-Pro	472.950 (9)	–0.3	0.54	–1.0	6.9
I-Ile	456.525 (10)	0.7	1.44	1.4	3.7
T-Thr	452.750 (11)	–0.2	1.01	–0.4	5.1
N-Asn	451.125 (12)	–0.5	0.59	–0.5	3.7
Q-Gln	435.025 (13)	–0.7	0.39	–1.1	4.7
W-Trp	434.575 (14)	0.3	1.06	1.6	0.7
L-Leu	426.700 (15)	0.5	1.36	0.5	7.4
E-Glu	404.075 (16)	–0.7	0.23	–1.3	6.5
R-Arg	384.875 (17)	–1.4	0.38	–0.7	8.7
S-Ser	368.500 (18)	–0.1	0.98	–0.7	8.8
A-Ala	359.750 (19)	0.3	1.26	0.2	8.3
K-Lys	336.275 (20)	–1.8 (20)	0.33 (18)	–1.6 (20)	7.9 (17)
*R*	1.00	0.532	0.415	0.478	–0.660

### Physicochemical Properties for BLPs

**Figure 4:**
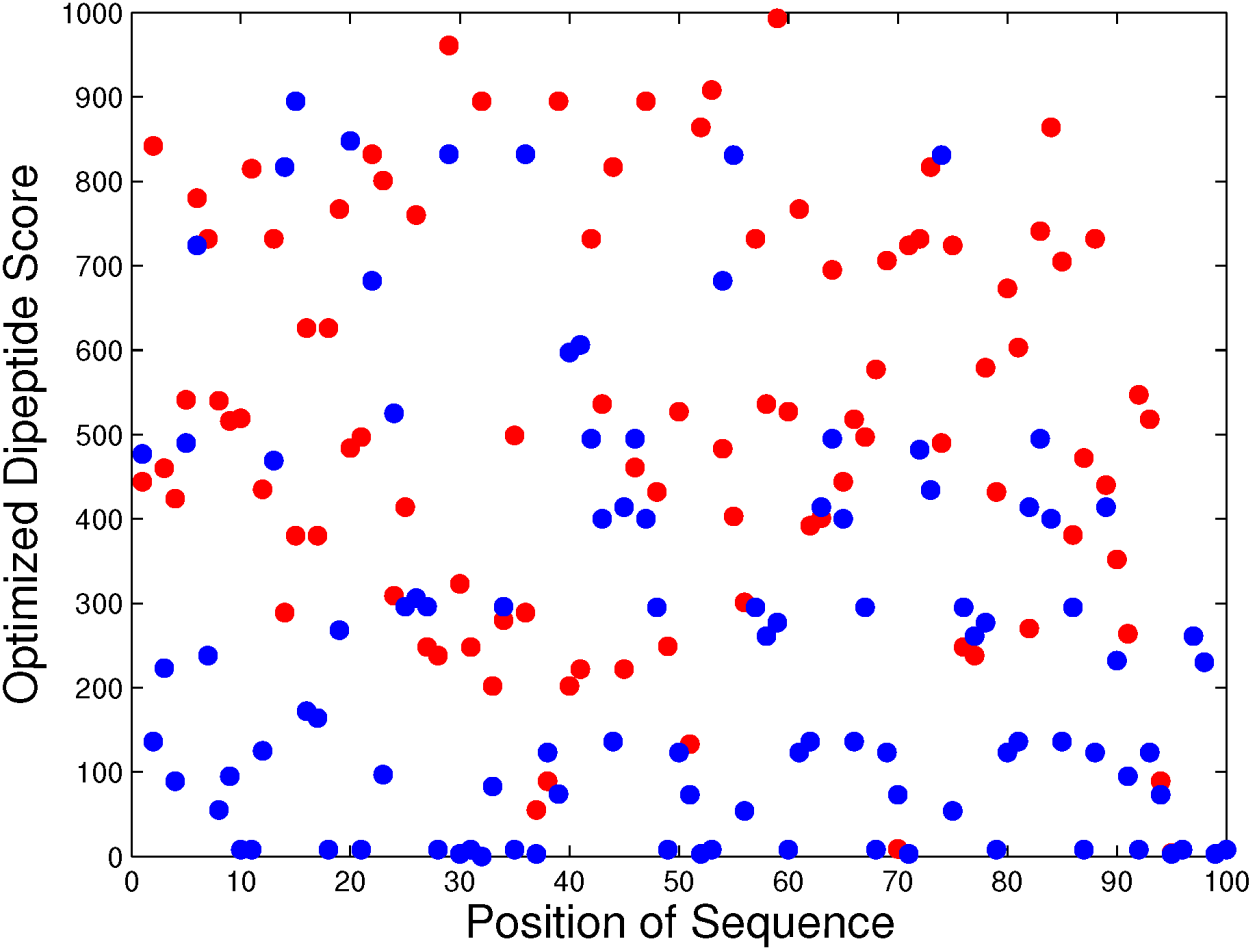
Distribution of dipeptide scores on two typical sequences of BLPs. The BLP with Pfam ID A8QZJ6 (length 96 with domain Oxidored shown in red) and the non-BLP with NCBI sequence entry GI: 20137223 (length 101 shown in blue) have high and low sequence scores 551.65 and 256.86, respectively.

The propensity scores come from the statistics of whole sequences to discriminate between BLPs and non-BLPs. Figure 4 shows the distribution of dipeptide scores on two typical sequences of BLP (Pfam ID A8QZJ6 of length 96 with domain Oxidored) and non-BLP (NCBI sequence entry GI: 20137223 of length 101) with high and low sequence scores of 551.65 and 256.86, respectively. This finding suggests that both high- and low-score dipeptides are evenly distributed on the sequences. Moreover, there are more high-score dipeptides in the BLP than in the non-BLP. Distribution of the dipeptide scores indicates no obviously high-score segment in the BLP. The above finding that top-ranked dipeptides do not tend to cluster in a certain region suggests that bioluminescence and fluorescence are a global property of BLP sequences.

1. The property of free energy of transfer (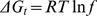) from the inside to the protein surface in globular proteins [20] has *R* = 0.532. Variable *f* of each amino acid is a ratio of the molar fractions of the buried and accessible residues. For example, amino acid Cys has the largest free energy of transfer 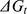
0.9 with *f* = 4.6, in which the molar fractions of the buried and accessible residues are 4.1 and 0.9, respectively. The propensity score of Cys is 572.85 at rank 2, which is quite close to 573.025 of Gly at rank 1. The residue with the smallest transfer free energy−1.8 is Lys at rank 20. This finding is consistent with a situation in which Lys has the smallest propensity score of 336.275 at rank 20. The residues with high propensity scores to be a BLP tend to be buried with a high free energy of transfer.
2. The property of average scale of membrane preference (AMP07) [21] has *R* = 0.415. The membrane-preference value for the amino acid residue j is defined as follows:

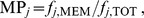
where *f_j_*_, MEM_ denotes the frequency of occurrence of residue j in the transmembrane regions of the proteins and *f_j_*_, TOT_ represents the frequency of occurrence of residue j along the entire sequences of the proteins, i.e. the molar fraction of residue j. Average scale of the membrane preference AMP07 has been computed as the arithmetical mean of seven different scales of the membrane preference. Residue Cys has the largest scale of membrane preference 1.60 at rank 1. Residue Lys having the smallest propensity score has the scale of membrane preference 0.33 at rank 18. Residues with high propensity scores to be a BLP tend to have residues with a high frequency of occurrence in the transmembrane regions. Consider the BLP with Pfam ID A8QZJ6 as an example. Three segments are in the transmembrane regions, which are in the positions 6∼22, 29∼50, and 56∼78, annotated in the Pfam database [14].

1. The property of hydrophobicity scale from the native protein structures of globular proteins [22] has *R* = 0.478. The structure-derived hydrophobic interaction along can distinguish a substantial number of native conformation from a large pool of misfolded structures. The hydrophobic interactions largely contribute to the stability of native folds, which agrees with experimental findings. Residue Cys has the largest hydrophobicity scale 1.9, which ranks 1^st^. The cysteine pair appears to be strongly hydrophobic since the formation of disulphide bonds increases the hydrophobicity of the reactants [23]. Residue Lys with the smallest propensity score has the smallest hydrophobicity scale −1.6 at rank 20. The hydrophobic potential is an important stabilizing interaction that is closely related to the native conformation. Moreover, the large propensity scores assigned to the hydrophobic residues agree with the hydrophobic characterization of luciferins, chromophore, and substrate-binding cavity [19].
2. The property of composition of amino acids in nuclear proteins [24] has *R*  =  −0.660. Correlation analysis of the amino acid composition and the cellular location of a protein is performed, which discriminates among the following five protein classes: integral membrane proteins, anchored membrane proteins, extracellular proteins, intracellular proteins and nuclear proteins [24]. BLPs tend to have a dislike of the amino acid composition of nuclear proteins, compared to that of integral membrane proteins.

Both integral membrane proteins and nuclear proteins were more closely examined by analyzing the compositions of BLPs, integral membrane proteins, and nuclear proteins, and their corresponding *R* values were analyzed, as shown in Table 5. The correlation coefficient between the compositions of BLPs and integral membrane proteins was *R* = 0.921, which is larger than *R* = 0.766 for nuclear proteins. The mean differences of composition of every amino acid were 0.77% and 1.31% for integral membrane proteins and nuclear proteins, respectively. Membrane proteins are rich in hydrophobic residues, which correspond to proteins having several transmembrane stretches of a secondary structure and poor in charged residues [24]. As is well recognized, the function of a protein is correlated with its subcellular localization, because the environment of a protein provides a portion of the relevant circumstance necessary for function. From the above analysis of the four properties, we hypothesize that BLPs tend to locate themselves near the cell membrane in order to execute their functions such as the emission of visible light (i.e. appropriate for the photic environment to surface regions) and receipt of extracellular ions for luciferases and photoproteins to trigger the bioluminescence reaction. Residue Cys in BLPs has the largest hydrophobicity scale and highest transfer free energy, which tends to be a buried residue. Consequently, the Cys composition was small, i.e. 2.09%, which ranked 19^th^ (i.e. larger than that of residue Trp). The above results support the analysis derived from the propensity scores of the SCMBLP method.

**Table 5:** The compositions of BLPs, integral membrane proteins, and nuclear proteins, and their corresponding *R* values using the training dataset BLP-TRN.

Amino acid	Composition of BLPs (%)	Composition of membrane (%)	Composition of nuclear (%)
G-Gly	7.53	7.0	6.3
C-Cys	2.09	2.0	1.6
F-Phe	4.58	5.6	2.7
H-His	2.75	2.0	2.1
Y-Tyr	3.56	3.3	2.4
D-Asp	5.82	3.8	4.7
M-Met	2.42	2.8	2.3
V-Val	6.60	7.7	5.3
P-Pro	4.99	4.7	6.9
I-Ile	5.60	6.7	3.7
T-Thr	5.51	5.6	5.1
N-Asn	4.23	3.7	3.7
Q-Gln	3.70	3.1	4.7
W-Trp	1.22	1.8	0.7
L-Leu	9.31	11.0	7.4
E-Glu	6.25	4.6	6.5
R-Arg	4.96	4.6	8.7
S-Ser	6.61	7.3	8.8
A-Ala	7.12	8.1	8.3
K-Lys	5.14	4.4	7.9
*R*	1.00	0.921	0.766

### Propensity Scores of Using SCMLFP

The propensity score of a sequence obtained using the prediction method SCMLFP is useful for discrimination between luciferases and FPs. The SCMLFP method uses the whole dataset consisting of 269 luciferases and 216 FPs to estimate the propensity scores of 400 dipeptides to be a luciferase against FP, as shown in Fig. 6. The training accuracy is 98.56%, and the sensitivity and specificity are 98.51% and 98.61%, respectively. Table 6 lists the propensity scores of 20 amino acids to be a luciferase against FP. For closely examining the different properties between luciferases and FPs, this table also shows the compositions of luciferases and FPs, composition difference between luciferases and FPs, and composition of integral membrane proteins. The total number of amino acids in the used luciferases and FPs is 107,742 and 133,473, respectively.

**Figure 5:**
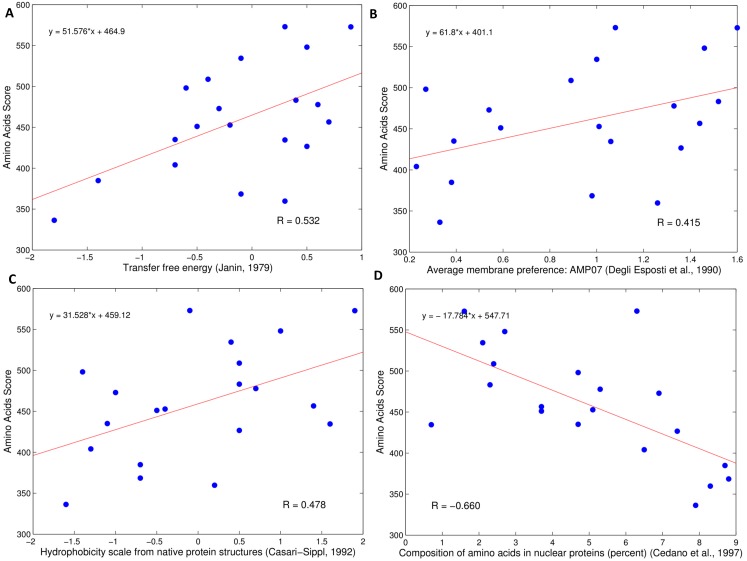
The correlation coefficients between the propensity scores and various physicochemical properties of 20 amino acids. (A) Transfer free energy (B) Average membrane preference (C) Hydrophobicity scale from native protein structures (D) Composition of amino acids in nuclear proteins.

**Figure 6:**
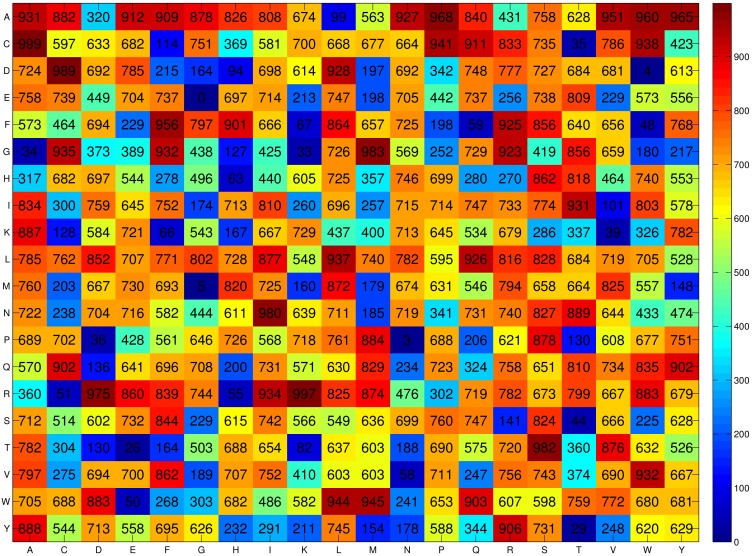
Heat map of the propensity scores of 400 dipeptides obtained from the SCMLFP method. Color bar is obtained using Jet of Matlab.

**Table 6:** The propensity scores of amino acids for distinguishing luciferases from FPs.

Amino acid	Propensity score	Composition of luciferases: A (%)	Composition of FPs: B (%)	Composition difference: A–B (%)	Composition of membrane (%)
L-Leu	729.900	10.41	7.17	3.24	11.0
A-Ala	726.425	8.25	5.04	3.21	8.1
R-Arg	674.050	5.64	3.75	1.89	4.6
S-Ser	650.575	6.61	5.23	1.38	7.3
I-Ile	646.125	6.05	4.78	1.27	6.7
Q-Gln	610.950	3.55	3.14	0.41	3.1
W-Trp	604.525	1.24	0.98	0.26	1.8
C-Cys	598.400	1.58	1.36	0.22	2.0
V-Val	594.625	6.52	6.45	0.07	7.7
F-Phe	591.925	4.82	4.89	–0.07	5.6
P-Pro	579.100	4.48	4.83	–0.35	4.7
N-Asn	575.950	3.85	4.22	–0.37	3.7
D-Asp	574.025	5.79	6.25	–0.46	3.8
E-Glu	569.000	6.34	6.85	–0.51	4.6
M-Met	555.800	2.47	3.31	–0.84	2.8
Y-Tyr	549.950	3.38	4.33	–0.95	3.3
T-Thr	535.050	4.84	6.21	–1.37	5.6
H-His	516.425	2.49	4.22	–1.73	2.0
G-Gly	490.975	6.84	9.31	–2.47	7.0
K-Lys	476.225	4.83	7.69	–2.86	4.4
*R*	1.00	0.503	–0.237	0.9996	0.522

The total number of amino acids in the used luciferases and FPs is 107,742 and 133,473, respectively.

The correlation coefficient between the propensity scores to be a luciferase and the compositions of luciferases and FPs are *R* = 0.503 and −0.237, respectively. A higher correlation of luciferases than that of FPs in terms of the absolute value of *R* reveals that the propensity scores characterize luciferases more appropriately. The two residues Leu and Ala have the highest scores, i.e. 729.900 and 726.425, which also have the largest percentages of composition in the luciferases, i.e. 10.41% and 8.25%, respectively. Notably, the top-two residues with the largest percentages (11.0% and 8.1%) of composition of residues in the integral membrane proteins are Leu and Ala, too. Leu and Ala are abundant in the transmembrane regions of the membrane proteins [21]. The hydrophobic residue Leu is frequently observed in transmembrane proteins [25].

The correlation coefficient between the propensity scores and the composition difference between luciferases and FPs is *R* = 0.9996. According to Eq. (1), the high correlation indicates that the estimated dipeptide scores can distinguish luciferases from FPs by simultaneously maximizing the prediction accuracy in terms of AUC and maximizing the correlation between the optimized and initial propensity scores of 20 amino acids (i.e. composition difference between luciferases and FPs).

The correlation coefficient between the propensity scores to be a luciferase and the composition of integral membrane proteins was *R* = 0.522. This study also examines the difference between luciferases and FPs from the aspect of composition. The correlation coefficients between the compositions of integral membrane proteins and those of luciferases and FPs were *R* = 0.937 and 0.635, respectively. The amino acid composition is commonly used as an informative feature when predicting subcellular localization [26]. Despite the inability of the amino acid composition to define protein location, a correlation is made between composition and location. We hypothesize that luciferases prefer a location near the cell membrane location rather than FPs for convenient receipt of extracellular ions.

### Prediction Accuracy of SCMLFP

The correlation coefficient between the compositions of luciferases and FPs was *R* = 0.72. The high value of *R* reveals that the prediction method using the feature of amino acid composition only cannot distinguish luciferases from FPs accurately. The SCMBLP method optimizing the propensity scores of 400 dipeptides by maximizing prediction accuracy is more effective in predicting BLPs than when using the SVM-based methods. Table 7 summarizes the classification accuracy of SCMLFP using 400 dipeptide scores from 10 independent runs on the training (LFP-TRN) and test (LFP-TEST) datasets. The training accuracy was 97.10±0.38% using LFP-TRN. The independent test performance was 96.28±3.35% with a sensitivity of 98.87±1.81% and a specificity of 93.01±7.37% on the LFP-TEST consisting of 26 luciferases and 22 FPs. This study also implemented the SVM-based methods using amino acid composition (SVM-AAC) and dipeptide composition (SVM-DPC) for comparisons; their test accuracies were 93.18±4.80% and 96.08±4.41%, respectively. Above results demonstrate that SCMLFP is better than SVM-AAC and comparable to SVM-DPC.

**Table 7:** The performance (%) of SCMLFP and the compared SVM-based methods on the dataset consisting of 269 luciferases and 216 FPs.

Method	Training Accuracy (%)	Test Accuracy (%)	Test Sensitivity (%)	Test Specificity (%)
SCMLFP	97.10±0.38	96.28±3.35	98.87±1.81	93.01±7.37
SVM-AAC	96.68±0.62	93.18±4.80	95.88±4.84	89.87±7.68
SVM-DPC	96.49±0.48	96.08±4.41	99.26±2.34	92.14±8.14

There are 10 independent runs on the training (LFP-TRN) and test (LFP-TEST) data sets.

Based on the above analysis of propensity scores of amino acids for luciferases and FPs, as is expected, the propensity scores of dipeptides can distinguish luciferases from FPs satisfactorily from the following two aspects. From the aspect of the classification mechanism, the residues and dipeptides with high propensity scores make up luciferases with large percentages of composition. Therefore, as is widely recognized, the weighted-sum propensity score of a sequence based on the SCM method can effectively distinguish luciferases from FPs. From the aspect of subcellular localization, the correlation between the compositions of luciferases and membrane proteins is as high as *R* = 0.937. The high correlation between the propensity scores and the composition of luciferases can characterize luciferases located near the membrane position.

### Illustrative Examples and Discussion

In this study, propensity scores are proposed to predict and characterize BLPs from primary sequences rather than informative crystal structures. Although the meaning of this apparently simple relationship (the *R* value) between the propensity scores and the identified physicochemical properties of amino acids is not very clear, additional comments are necessary to gain further insight into BLPs, including luciferases, photoproteins, and FPs. To characterize BLPs more accurately, this study discusses the propensity scores and the identified properties by using illustrative models with known structures of BLPs and illustrative applications of photoproteins.

**Figure 7:**
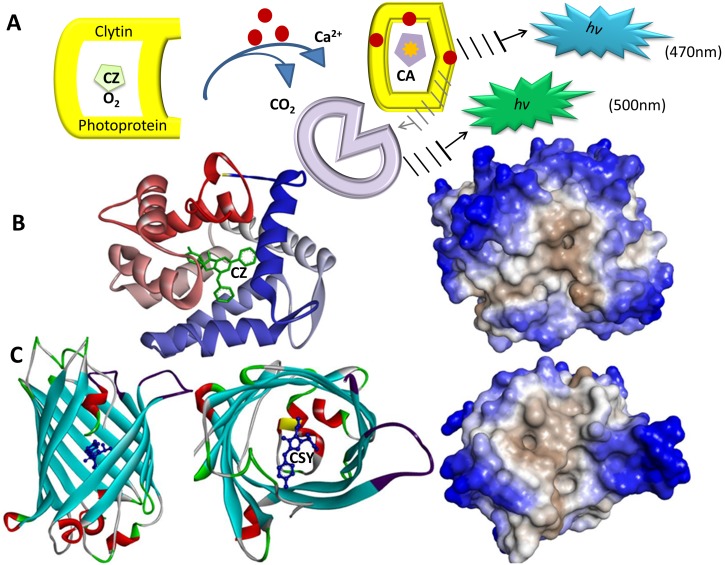
Bioluminescence system of the jellyfish *Clytia* with the GFP-clytin complex. (A) Clytin is a Ca^2+^-regulated photoprotein comprising coelenterazine (CZ) as a substrate for its bioluminescence reaction. When Ca^2+^ binds to clytin, the bioluminescence reaction is triggered to produce the excited state product coelenteramide (CA) and CO_2_ with emission of a broad blue bioluminescence (maximum 470 nm). Due to energy transfer, Clytia GFP fluorescence is in green (maximum 500 nm). (B) The structure of clytin (22.4 kDa, PDB code 3KPX) and its representation using hydrophilic (outer part in blue) and hydrophobic (inner part) residues. (C) The structure of Clytia GFP (PDB code 2HPW) and its representation using hydrophilic and hydrophobic residues using Discovery Studio 3.5. The visible fluorophore of Clytia GFP (CSY) is a sequence of three amino acids (68S, 69Y, and 70G). To protect the chromophore fluorescence from quenching by water, the tightly packed nature of the barrel excludes solvent molecules resulting in that the environment comprising this fluorophore is also hydrophobic.

Titushin *et al*. presented a review towards understanding the role of protein-protein interactions in the function of various bioluminescence systems [19]. That review provides valuable insight into the detailed mechanism of bioluminescence using the most reliable structural model which is available for the protein-protein complex of the Ca^2+^-regulated photoprotein clytin and GFP from the jellyfish *Clytia gocking* [19]. Figure 7 schematically depicts the bioluminescence system of the jellyfish *Clytia* with the GFP-clytin complex. Clytin shares a high structural and sequence similarity with the other Ca^2+^-regulated photoproteins such as aequorin from *Aequorea*. Clytin comprises the hydrophobic coelenterazine as a substrate for its bioluminescence reaction, as shown in Fig. 7(A). When Ca^2+^ binds to the hydrophobic substrate-binding cavity of clytin, the bioluminescence reaction is triggered to produce the excited state product coelenteramide and CO_2_ with emission of a broad blue bioluminescence (maximum 470 nm). Clytia GFP has a low sequence identity and a structure that is highly similar to those of GFPs from *Aequorea* [27]. Owing to the energy transfer, Clytia GFP extends the bioluminescence to slightly longer wavelengths; the resulting fluorescence is in green (maximum 500 nm). Closely examining the structure of the GFP-clytin complex reveals that the complexation is governed by several hydrophobic contacts and a hydrogen bond network [19]. The SCMBLP and SCMLFP methods predicted the photoprotein clytin and obtained the propensity scores of 463.63 and 572.16, respectively. The threshold values to classify BLPs and luciferases are 439.63 and 577.12, respectively. According to the prediction results, clytin is a BLP with high confidence and a FP with low confidence (the sequence score close to the threshold value). Additionally, the SCMBLP and SCMLFP methods predicted the Clytia GFP and obtained the propensity scores of 464.98 and 560.94, respectively. The prediction result was accurate that Clytia GFP was categorized into BLP and FP with high confidence.

Figure 7(B) shows the structure of clytin (PDB code 3KPX) and its representation using hydrophilic and hydrophobic residues. This figure reveals the hydrophobic coelenterazine and the hydrophobic substrate-binding cavity of clytin. Similarly, Fig. 7(C) shows a visible fluorophore of Clytia GFP (CSY) from a sequence of three amino acids (68S, 69Y, and 70G). To protect the chromophore fluorescence from quenching by water, the tightly packed nature of the barrel excludes solvent molecules resulting in that the environment comprising this fluorophore is also hydrophobic.

Based on Fig. 7 and the prediction results, we can infer the following. First, the propensity scores of sequences can classify the clytin and Clytia GFP into BLPs accurately. The SCMLFP method was designed using the training datasets consisting of luciferases and FPs. Therefore, the propensity score of the photoprotein clytin was close to the decision threshold value. Luciferases (oxidative enzymes) have higher average propensity scores of using SCMLFP than photoproteins. Second, the hydrophobic coelenterazine and substrate-binding cavity of clytin, as well as the hydrophobic contacts in the protein-protein interaction in the GFP-clytin complex support the identified physicochemical properties. Notably, the residues of BLPs with high propensity scores tend to be buried with high transfer free energy and hydrophobic.

**Figure 8:**
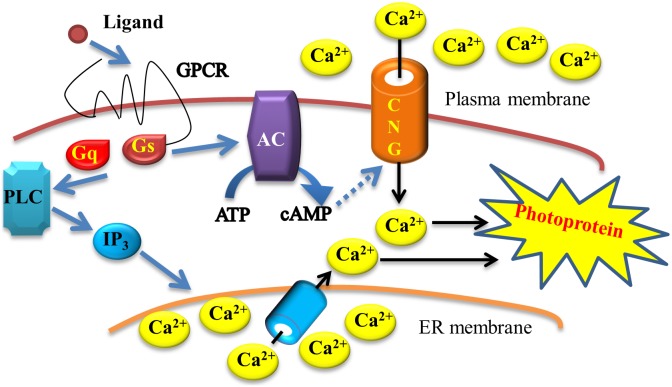
Schematic presentation of the G protein-coupled receptor (GPCR) reporter cell line for drug discovery. The drug discovery of high-throughput screening is monitored by activation of the calcium-sensitive photoprotein light production. Ligand-mediated activation of Gs-coupled receptors stimulates cAMP synthesis by adenylyl cyclase (AC) and opening of the cAMP-gated heteromultimeric cyclic nucleotide-gated (CNG) channel. Calcium ions from the extracellular solution enter the cell through the CNG channel and are detected by photoprotein luminescence measurements. Gq-coupled receptor activation stimulates the phospholipase C (PLC)/IP3 pathway detected via photoprotein luminescence stimulated by calcium released from the endoplasmic reticulum (ER).

Owing to the easy detection of the emitted light with an illuminometer, photoproteins are widely used in molecular biology to measure intracellular Ca^2+^ levels. The G protein-coupled receptor (GPCR) family is the largest known class of molecular targets with a proven therapeutic value. Cell surface GPCRs drive numerous signaling pathways involved in the regulation of a broad range of physiological processes [28]. GPCRs can couple with Ca^2+^ signaling in the drug discovery of high-throughput screening. Photoproteins are also increasingly used to detect activation of other molecular target classes such as ligand-gated ion channels and transporters [29]. Figure 8 schematically depicts the GPCR reporter cell line for drug discovery monitored by activation of the calcium-sensitive photoprotein light production [30]. Ligand-mediated activation of Gs-coupled receptors stimulates cAMP synthesis by adenylyl cyclase (AC) and opening of the cAMP-gated heteromultimeric cyclic nucleotide-gated (CNG) channel. While entering the cell through the CNG channel, Calcium ions from the extracellular solution are detected by photoprotein luminescence measurements. Gq-coupled receptor activation stimulates the phospholipase C (PLC)/IP3 pathway detected via photoprotein luminescence stimulated by calcium released from the endoplasmic reticulum (ER).

Conventionally, changes in intracellular Ca^2+^ levels have been readily detected using fluorescent dyes that emit light in proportion to changes in intracellular Ca^2+^ concentration. An alternative approach to measuring indirectly the changes in Ca^2+^ concentrations involves using recombinantly expressed biosensor photoproteins, of which aequorin is a prototypic example [31]. These biosensors provide an intense luminescent signal in response to elevations in intracellular Ca^2+^ [29]. Bovolenta *et al*. engineered a new photoprotein, Photina, to overcome some of the limitations of aequorin by exchanging a portion of the sequence of the photoprotein obelin [32] with a corresponding region in clytin to combine the best properties of each photoprotein [33]. The signal of Photina cells is approximately 3-folds higher than the signal of cells expressing aequorin in the mitochondria. Cainarca *et al*. stably expressed c-Photina, a Ca^2+^-sensitive photoprotein, driven by a ubiquitous promoter in a mouse embryonic stem cell line [34].

Photoproteins can detect both extracellular and intracellular Ca^2+^ influxes. For the receipt of extracellular magnesium or calcium ions, luciferases and photoproteins have a similar composition of integral membrane proteins. The residues with high propensity scores to be a luciferase tend to have residues with a high frequency of occurrence in the transmembrane regions. Photoprotein mutants and modified versions of coelenterazine can increase the range of the recorded calcium concentrations. The presented propensity scores and characterization of BLPs greatly facilitates mutagenesis analysis to enhance the bioluminescence and fluorescence of BLPs.

## Conclusions

Predicting and characterizing bioluminescent proteins (BLPs), including luciferases, photoproteins, and fluorescent proteins (FPs), are valuable for commercial and medical applications yet more challenging, owing to their high variety of protein sequences and small number of crystal structures. Given the increasing number of innovative tools in drug discovery based on engineered luciferases, photoproteins, and FPs, accurate and easily interpreted computational methods must be developed to predict and analyze BLPs. This study has proposed a scoring card method (SCM) based approach to estimate the propensity scores of 400 dipeptides and 20 amino acids from sequences in order to design two prediction methods (i.e. SCMBLP and SCMLFP) and characterize BLPs using some identified physicochemical properties. Analysis results indicate that the SCMBLP method performs better than the support vector machine (SVM) based prediction methods. By establishing the datasets of luciferases and FPs, to our knowledge, the proposed SCMLFP method is the first prediction method capable of distinguishing luciferases from FPs. The two sets of propensity scores to be BLPs and luciferases are highly promising for use in discovering new properties to more thoroughly elucidate BLPs. For example, the residues of BLPs with high propensity scores tend to be buried with a high transfer free energy and hydrophobic in nature. As for the high correlation between the compositions of luciferases and integral membrane proteins, we hypothesize that luciferases prefer a location near the cell membrane location for convenient receipt of extracellular ions. Furthermore, the propensity scores and identified properties for BLPs greatly facilitate mutagenesis analysis to enhance the bioluminescence and fluorescence of BLPs.

## Supporting Information

Table S1 **The used 39 bioluminescent proteins from Protein Data Bank (PDB).** The threshold value to discriminate between BLPs and non-BLPs is 439.627.
(DOCX)

Table S2 **The 94 bioluminescent proteins using the GO Term GO: 0008218 “bioluminescence” annotated on SwissProt.** The threshold value to discriminate between BLPs and non-BLPs is 439.627.
(DOCX)

File S1 **The used 269 luciferases.**
(XLS)

File S2 **The used 216 fluorescent proteins.**
(XLS)

## References

[1] Wilson T (1995) Comments on the Mechanisms of Chemi- and Bioluminescence. Photochemistry and Photobiology 62: 601–606.

[2] White EH, Rapaport E, Seliger HH, Hopkins TA (1971) The chemi- and bioluminescence of firefly luciferin: An efficient chemical production of electronically excited states. Bioorganic Chemistry 1: 92–122.

[3] Head JF, Inouye S, Teranishi K, Shimomura O (2000) The crystal structure of the photoprotein aequorin at 2.3 A resolution. Nature 405: 372–376. 10830969 10.1038/35012659

[4] Cubitt AB, Heim R, Adams SR, Boyd AE, Gross LA, et al (1995) Understanding, improving and using green fluorescent proteins. Trends Biochem Sci 20: 448–455. 8578587 10.1016/s0968-0004(00)89099-4

[5] Vidi PA, Watts VJ (2009) Fluorescent and bioluminescent protein-fragment complementation assays in the study of G protein-coupled receptor oligomerization and signaling. Mol Pharmacol 75: 733–739. 19141658 10.1124/mol.108.053819 PMC2684918

[6] Kandaswamy KK, Pugalenthi G, Hazrati MK, Kalies KU, Martinetz T (2011) BLProt: prediction of bioluminescent proteins based on support vector machine and relieff feature selection. BMC Bioinformatics 12: 345. 21849049 10.1186/1471-2105-12-345 PMC3176267

[7] Huang HL, Liou YF, Lee HC, Huang WL, Ho SY (2012) Designing predictors of bioluminescence proteins using an efficient physicochemical property mining method”, IEEE International Conference on Bioinformatics and Biomedical Engineering. iCBBE 2012 40–43.

[8] Zhao X, Li J, Huang Y, Ma Z, Yin M (2012) Prediction of bioluminescent proteins using auto covariance transformation of evolutional profiles. Int J Mol Sci 13: 3650–3660. 22489173 10.3390/ijms13033650 PMC3317733

[9] Fan GL, Li QZ (2013) Discriminating bioluminescent proteins by incorporating average chemical shift and evolutionary information into the general form of Chou’s pseudo amino acid composition. J Theor Biol 334: 45–51. 23770403 10.1016/j.jtbi.2013.06.003

[10] Huang HL, Charoenkwan P, Kao TF, Lee HC, Chang FL, et al (2012) Prediction and analysis of protein solubility using a novel scoring card method with dipeptide composition. BMC Bioinformatics 13 Suppl 17 S3. 10.1186/1471-2105-13-S17-S3 PMC3521471 23282103

[11] Charoenkwan P, Shoombuatong W, Lee HC, Chaijaruwanich J, Huang HL, et al (2013) SCMCRYS: predicting protein crystallization using an ensemble scoring card method with estimating propensity scores of P-collocated amino acid pairs. PLoS One 8: e72368. 24019868 10.1371/journal.pone.0072368 PMC3760885

[12] Huang HL, Lin IC, Liou YF, Tsai CT, Hsu KT, et al (2011) Predicting and analyzing DNA-binding domains using a systematic approach to identifying a set of informative physicochemical and biochemical properties. BMC Bioinformatics 12 Suppl 1 S47. 21342579 10.1186/1471-2105-12-S1-S47 PMC3044304

[13] Kawashima S, Pokarowski P, Pokarowska M, Kolinski A, Katayama T, et al (2008) AAindex: amino acid index database, progress report 2008. Nucleic Acids Res 36: D202–205. 17998252 10.1093/nar/gkm998 PMC2238890

[14] Sonnhammer EL, Eddy SR, Durbin R (1997) Pfam: a comprehensive database of protein domain families based on seed alignments. Proteins 28: 405–420. 9223186 10.1002/(sici)1097-0134(199707)28:3<405::aid-prot10>3.0.co;2-l

[15] Shinn-Ying H, Li-Sun S, Jian-Hung C (2004) Intelligent evolutionary algorithms for large parameter optimization problems. Evolutionary Computation, IEEE Transactions on 8: 522–541.

[16] Bradley AP (1997) The use of the area under the ROC curve in the evaluation of machine learning algorithms. Pattern Recognition 30: 1145–1159.

[17] Tung CW, Ho SY (2007) POPI: predicting immunogenicity of MHC class I binding peptides by mining informative physicochemical properties. Bioinformatics 23: 942–949. 17384427 10.1093/bioinformatics/btm061

[18] Kyte J, Doolittle RF (1982) A simple method for displaying the hydropathic character of a protein. J Mol Biol 157: 105–132. 7108955 10.1016/0022-2836(82)90515-0

[19] Titushin MS, Feng Y, Lee J, Vysotski ES, Liu ZJ (2011) Protein-protein complexation in bioluminescence. Protein Cell 2: 957–972. 22231355 10.1007/s13238-011-1118-y PMC4875246

[20] Janin J (1979) Surface and inside volumes in globular proteins. Nature 277: 491–492. 763335 10.1038/277491a0

[21] Degli Esposti M, Crimi M, Venturoli G (1990) A critical evaluation of the hydropathy profile of membrane proteins. Eur J Biochem 190: 207–219. 2364947 10.1111/j.1432-1033.1990.tb15566.x

[22] Casari G, Sippl MJ (1992) Structure-derived hydrophobic potential. Hydrophobic potential derived from X-ray structures of globular proteins is able to identify native folds. J Mol Biol 224: 725–732. 1569551 10.1016/0022-2836(92)90556-y

[23] Weast RC (1974) Editor of Handbook of Chemistry and Physics, 55th, CRC Press, Cleveland.

[24] Cedano J, Aloy P, Perez-Pons JA, Querol E (1997) Relation between amino acid composition and cellular location of proteins. J Mol Biol 266: 594–600. 9067612 10.1006/jmbi.1996.0804

[25] Nakashima H, Yoshihara A, Kitamura K (2013) Favorable and unfavorable amino acid residues in water-soluble and transmembrane proteins J. Biomedical Science and Engineering 6 36–44.

[26] Huang WL, Tung CW, Ho SW, Hwang SF, Ho SY (2008) ProLoc-GO: utilizing informative Gene Ontology terms for sequence-based prediction of protein subcellular localization. BMC Bioinformatics 9: 80. 18241343 10.1186/1471-2105-9-80 PMC2262056

[27] Ormo M, Cubitt AB, Kallio K, Gross LA, Tsien RY, et al (1996) Crystal structure of the Aequorea victoria green fluorescent protein. Science 273: 1392–1395. 8703075 10.1126/science.273.5280.1392

[28] Denis C, Saulière A, Galandrin S, Sénard JM, Galés C (2012) Probing heterotrimeric G protein activation: applications to biased ligands. Curr Pharm Des 18: 17. 10.2174/138161212799040466 PMC3389521 22229559

[29] Eglen RM, Reisine T (2008) Photoproteins: important new tools in drug discovery. Assay Drug Dev Technol 6: 659–671. 19035847 10.1089/adt.2008.160

[30] Wunder F, Rebmann A, Geerts A, Kalthof B (2008) Pharmacological and kinetic characterization of adrenomedullin 1 and calcitonin gene-related peptide 1 receptor reporter cell lines. Mol Pharmacol 73: 1235–1243. 18174292 10.1124/mol.107.042283

[31] Bonora M, Giorgi C, Bononi A, Marchi S, Patergnani S, et al (2013) Subcellular calcium measurements in mammalian cells using jellyfish photoprotein aequorin-based probes. Nat Protoc 8: 2105–2118. 24113784 10.1038/nprot.2013.127

[32] Campbell AK (1974) Extraction, partial purification and properties of obelin, the calcium-activated luminescent protein from the hydroid Obelia geniculata. Biochem J 143: 411–418. 4156828 10.1042/bj1430411 PMC1168396

[33] Bovolenta S, Foti M, Lohmer S, Corazza S (2007) Development of a Ca(2+)-activated photoprotein, Photina, and its application to high-throughput screening. J Biomol Screen 12: 694–704. 17517900 10.1177/1087057107301497

[34] Cainarca S, Fenu S, Ferri C, Nucci C, Arioli P, et al (2010) A photoprotein in mouse embryonic stem cells measures Ca2+ mobilization in cells and in animals. PLoS One 5: e8882. 20111708 10.1371/journal.pone.0008882 PMC2811732

