# Supplementary material for: Propensity Scores for Prediction and Characterization of Bioluminescent Proteins from Sequences
Source: PLoS One. 2014 May 14;9(5):e97158. doi: 10.1371/journal.pone.0097158 (PMC4020813; doi:10.1371/journal.pone.0097158)
Supplement: Table S1 — The used 39 bioluminescent proteins from Protein Data Bank (PDB). The threshold value to discriminate between BLPs and non-BLPs is 439.627. (DOCX) [file pone.0097158.s001.docx]

**Table S1 The used 39** [**bioluminescent**](http://en.wikipedia.org/wiki/Bioluminescent) [**protein**](http://en.wikipedia.org/wiki/Proteins)**s from Protein Data Bank (PDB).** The threshold value to discriminate between BLPs and non-BLPs is 439.627.

| PDB ID | Score | PDB ID | Score | PDB ID | Score | PDB ID | Score |
| --- | --- | --- | --- | --- | --- | --- | --- |
| 1BA3 | 448.44 | 2HPS | 448.97 | 2WSO | 489.50 | 3G9A | 476.14 |
| 1BSL | 429.75 | 2HQ8 | 448.59 | 2WUR | 490.09 | 3GEX | 487.95 |
| 1EJ3 | 438.03 | 2QRF | 488.37 | 3CGL | 486.24 | 3GJ1 | 483.72 |
| 1EL4 | 473.27 | 2QT2 | 491.08 | 3DPW | 493.19 | 3IEP | 448.06 |
| 1JF0 | 475.41 | 2QU1 | 489.90 | 3E5T | 473.22 | 3K1K | 468.14 |
| 1JF2 | 475.05 | 2QZ0 | 488.07 | 3E5V | 475.93 | 3KPX | 463.63 |
| 1QV0 | 474.14 | 2V4E | 491.08 | 3E5W | 466.86 | 3LA1 | 490.43 |
| 1SL7 | 472.65 | 2VAD | 485.08 | 3EK4 | 465.52 | 4H48 | 488.10 |
| 1SL8 | 441.83 | 2VAE | 486.27 | 3EVP | 491.20 | 4MN0 | 447.64 |
| 2F8P | 474.15 | 2WSN | 487.21 | 3FGC | 446.31 |  |  |
