# Supplementary material for: Propensity Scores for Prediction and Characterization of Bioluminescent Proteins from Sequences
Source: PLoS One. 2014 May 14;9(5):e97158. doi: 10.1371/journal.pone.0097158 (PMC4020813; doi:10.1371/journal.pone.0097158)
Supplement: Table S2 — The 94 bioluminescent proteins using the GO Term GO: 0008218 “bioluminescence” annotated on SwissProt. The threshold value to discriminate between BLPs and non-BLPs is 439.627. (DOCX) [file pone.0097158.s002.docx]

**Table S2 The 94** [**bioluminescent**](http://en.wikipedia.org/wiki/Bioluminescent) [**protein**](http://en.wikipedia.org/wiki/Proteins)**s using the GO Term GO:0008218 “bioluminescence” annotated on SwissProt.** The threshold value to discriminate between BLPs and non-BLPs is 439.627.

| Entry | Score |  | Entry | Score |  | | Entry | Score |  | | Entry | Score |
| --- | --- | --- | --- | --- | --- | --- | --- | --- | --- | --- | --- | --- |
| P07164 | 447.36 |  | Q27709 | 448.44 |  | P19840 | | 442.51 |  | P12748 | | 444.70 |
| P02592 | 441.04 |  | Q9GV45 | 438.15 |  | P23147 | | 465.04 |  | Q9S3Z2 | | 452.22 |
| P42212 | 493.05 |  | P08659 | 477.46 |  | P23113 | | 443.49 |  | P24272 | | 438.70 |
| Q9U6Y6 | 494.05 |  | P09140 | 428.56 |  | P19197 | | 439.93 |  | P24273 | | 453.22 |
| Q9GZ28 | 472.43 |  | P29238 | 459.17 |  | P23148 | | 441.55 |  | P12747 | | 477.46 |
| P0DM59 | 423.86 |  | P09141 | 456.48 |  | P19842 | | 456.48 |  | P12746 | | 459.17 |
| Q9U6Y3 | 459.24 |  | P29239 | 466.83 |  | Q7N575 | | 454.56 |  | P21578 | | 452.29 |
| Q08121 | 481.47 |  | Q03324 | 445.56 |  | Q7N574 | | 456.51 |  | Q5DZ04 | | 456.74 |
| Q95W86 | 476.75 |  | P29236 | 430.20 |  | Q7N577 | | 457.68 |  | P35328 | | 450.90 |
| Q95W11 | 480.42 |  | P21309 | 453.78 |  | Q7N576 | | 466.74 |  | P35327 | | 447.65 |
| Q9U6Y8 | 491.15 |  | Q06878 | 455.18 |  | Q27757 | | 455.21 |  | B5EV70 | | 448.68 |
| Q9U6Y7 | 485.32 |  | Q9U6Y5 | 453.78 |  | P05938 | | 445.71 |  | P43127 | | 471.15 |
| P39047 | 471.15 |  | P09142 | 427.39 |  | P27652 | | 446.10 |  | Q56691 | | 472.43 |
| Q8ISF8 | 478.49 |  | P29237 | 452.29 |  | Q9AJA7 | | 450.44 |  | P07740 | | 476.75 |
| Q95P04 | 490.52 |  | Q06877 | 448.68 |  | B1KD61 | | 452.51 |  | P07739 | | 480.42 |
| Q95W85 | 486.15 |  | P24113 | 439.98 |  | C6KYS2 | | 473.80 |  | P08639 | | 490.52 |
| P18299 | 441.98 |  | P12744 | 431.10 |  | P17554 | | 470.38 |  | P05521 | | 486.15 |
| P18300 | 441.39 |  | P19841 | 460.86 |  | P24114 | | 448.68 |  | P14286 | | 498.51 |
| O77206 | 448.51 |  | P41302 | 429.75 |  | Q9KT83 | | 407.44 |  | P16447 | | 475.41 |
| P13129 | 445.46 |  | P12745 | 473.80 |  | Q9U6Y4 | | 423.86 |  | P21308 | | 473.27 |
| Q01158 | 498.51 |  | P25082 | 456.74 |  | P46072 | | 458.05 |  | A7N6K1 | | 491.15 |
| Q26304 | 475.41 |  | P43129 | 484.59 |  | P43126 | | 438.15 |  | P43128 | | 478.49 |
| P83690 | 473.27 |  | P19839 | 492.40 |  | P19907 | | 428.56 |  |  | |  |
| Q8T6Z0 | 458.23 |  | P23146 | 435.59 |  | P19908 | | 449.16 |  |  | |  |
